# ACEI/ARB and beta-blocker therapies for preventing cardiotoxicity of antineoplastic agents in breast cancer: a systematic review and meta-analysis

**DOI:** 10.1007/s10741-023-10328-z

**Published:** 2023-07-07

**Authors:** Yu Gao, Ruiting Wang, Jinchi Jiang, Yueyao Hu, Haijing Li, Yong Wang

**Affiliations:** 1 College of Chinese Medicine, Beijing University of Chinese Medicine, Beijing, 100029 China; 2 Graduate School, Beijing University of Chinese Medicine, Beijing, 100029 China; 3 School of Life Sciences, Beijing University of Chinese Medicine, Beijing, 100029 China

**Keywords:** Cardiotoxicity, LVEF, Breast cancer, ACEI/ARB, Beta-blocker

## Abstract

Anthracyclines and trastuzumab are widely used to treat breast cancer but increase the risk of cardiomyopathy and heart failure. With the use of trastuzumab and anthracycline-containing medications, this study intends to evaluate the effectiveness and security of current treatments against cardiotoxicity. We conducted a systematic review of randomized controlled trials (RCTs), which used at least one angiotensin-converting enzyme inhibitor (ACEI), angiotensin receptor blocker (ARB), or beta-blocker (BB) to prevent cardiotoxicity of antineoplastic agents for breast cancer, in 4 databases (PubMed, Cochrane Library, EMBASE, Web of Science) from inception to 11 May 2022, without language restrictions. The outcome of interest was left ventricular ejection fraction (LVEF) and adverse events. Stata 15 and R software 4.2.1 were used to perform all statistical analyses. The Cochrane version 2 of the risk of bias tool was used to assess the risk of bias, and the grading of recommendations assessment, development, and evaluation (GRADE) assessment was used to appraise the quality of the evidence. Fifteen randomized clinical studies with a total of 1977 patients were included in the analysis. The included studies demonstrated statistically significant LVEF in the ACEI/ARB and BB treatment groups (*χ*^2^ = 184.75, *I*^2^ = 88.6%, *p* = 0.000; SMD 0.556, 95% CI 0.299 to 0.813). In an exploratory subgroup analysis, the benefit of experimental agents on LVEF, whether anthracyclines or trastuzumab, was prominent in patients treated with ACEIs, ARBs, and BBs. Compared to placebo, ACEI/ARB and BB treatments in breast cancer patients protect against cardiotoxicity after trastuzumab and anthracycline-containing medication treatment, indicating a benefit for both.

**Supplementary Information:**

The online version contains supplementary material available at 10.1007/s10741-023-10328-z.

## Introduction

Cancer is one major cause of morbidity and mortality worldwide, and in response, anticancer therapies are increasing to prolong the survival of patients [1]. In comparison, the cardiotoxicity associated with anticancer therapy poses a challenge for oncologists and cardiologists [2]. Of these, anthracyclines and trastuzumab are widely used to treat breast cancer, the leading cause of death from cancer in women, but are associated with several cardiovascular toxicities [3–5]. The latest oncology cardiology guidelines recommend angiotensin-converting enzyme inhibitor (ACEI), angiotensin receptor blocker (ARB), beta-blocker (BB), and calcium channel blocker (CCB) classes as cardioprotective drugs [6]. Although ACEIs, ARBs, and BBs have been investigated as primary prevention options, the strength of the available evidence does not support using these medications routinely for breast cancer patients receiving anthracyclines and/or trastuzumab [7, 8]. Currently, it is not clear which drug is the best choice as the primary option. Incorporating various recent studies into an updated meta-analysis can significantly improve the current body of evidence available. The purpose of this review is to thoroughly analyze studies that specifically focus on the heart-protective benefits of ACEI/ARBs and BBs in breast cancer patients who have undergone treatment with anthracycline or trastuzumab.

## Methods

The PRISMA Checklist 2020 was used to perform this study (Supplemental Table 1) [9].

**Table 1:** Baseline characteristics of the selected randomized controlled trial

Study ID	Primary study drug	Cancer stage	No. of patients	Group (no. of patients)	Age (years)	Female sex (%)	Duration of anticancer therapy (months)	Type and dosage of ACEI/ARB or BB	Duration of ACEI/ARB or BB (months)	Follow-up (months)	Outcome	Type Of LVEF measurement	Mean baseline EF (%)
Livi et al. 2021 [12]	Anthracycline	Early stage	174	Placebo (42), ACEI (44), BB (45), both (43)	48 ± 25.5	100%	12	Bisoprolol (5 mg Qd), ramipril (5 mg Qd)	12	24	LVEF, GLS	Echo	66.0 to 68.5, 64.7 to 66.7, 64.7 to 66.7, 65.5 to 67.4
Lee et al. 2021 [14]	Anthracycline	I, II, III, IV	195	Control (43), ACEI/ARB (82), BB (70)	48.5 ± 10.4, 47.8 ± 8.7, 46.6 ± 7.6	100%	3	Candesartan (4 mg Qd), carvedilol (3.125 mg Qd)	2	12	LVEF, LA size, LV size, adverse events	Echo	66.9 ± 4.5, 67.9 ± 1.8, 68.7 ± 5.2
Wihandono et al. 2021 [13]	Anthracycline	Locally advanced IIIB IIIC	51	Control (25), both (26)	50.8 ± 7.39, 44.5 ± 7.78	100%	6 cycles	Lisinopril (2.5–10 mg Qd), bisoprolol (2.5–10 mg Qd)	42	6	LVEF, adverse events	Echo	65.64 ± 4.55, 65.77 ± 4.56
Guglin et al. 2020 [16, 27, 28]	Trastuzumab	Early stage	468	Control (154), ACEI (158), BB (156)	51 ± 10.7	100%	12	Lisinopril, carvedilol	12	24	LVEF	Echo.MUGA	62.24 ± 6.09, 62.97 ± 6.18, 62.55 ± 6.17
Esfandbod et al. 2021 [15]	Trastuzumab	Early stage	60	Control (30), BB (30)	46.2 ± 8.59, 47.6 ± 9.64	100%	16	Carvedilol (3.125–12.5 mg Bid)	23	12	EF and PAP	Echo	54.9 ± 0.45, 55 ± 1.03
Avila et al. 2018 [19]	Anthracycline	NA	192	Placebo (96), BB (96)	53 ± 9	100%	6	Carvedilol (3.125–25 mg Bid)	4	6	LVEF, BNP, TNI, adverse events	Echo	65.2 ± 3.6, 64.8 ± 4.7
Cochera et al. 2018 [18]	Anthracycline	NA	60	Control (30), BB (30)	53 ± 13	100%	4.5	Nebivolol (5 mg Qd)	4.5	EOT	LVEF, FS, and LV Diameters	Echo	61.0 ± 2, 62.0 ± 4
Moshkani et al. 2019 [17]	Trastuzumab	Early stage	71	Control (35), BB (36)	57.4 ± 8.8, 57.3 ± 7.3	100%	3	Carvedilol (6.25–25 mg Bid)	3	3	GLS, SRS, adverse events	Echo	54.32 ± 5.32, 54.93 ± 4.26
Nabati et al. 2017 [21]	Anthracycline	NA	91	Control (45), BB (46)	47.1 ± 12	100%	105	Carvedilol (3.125–6.125 mg Bid)	6	EOT (6)	LVEF, adverse events	Echo	61.0 ± 4, 58.7 ± 4
Pituskin et al. 2017 [20]	Trastuzumab	Early stage	94	Placebo (30), ACEI (33), BB (31)	53 ± 10	100%	12	Perindopril (2 mg Qd), bisoprolol (2.5 mg Qd)	12	After trast (12)	LVEF, adverse events	MRI	61 ± 5, 61 ± 4, 62 ± 5
Tashakori et al. 2016 [22]	Anthracycline	Early stage	70	Placebo (40), carvedilol (30)	39.9, 42	100%	1.5	Carvedilol (6.25 mg Qd)	1.5	EOT	LVEF, strain, strain-rate parameters	Echo	59.41 ± 4.2, 61.31 ± 3.2
Boekhout et al. 2016 [24]	Trastuzumab	Early stage	206	Placebo (103), ARB (103)	50 ± 7	100%	12	Candesartan (16–32 mg Qd)	18	21	LVEF, adverse events	Echo.MUGA	60.7 ± 2.5, 63.2 ± 6.5
Gulati et al. 2016 [23]	Anthracycline	Early stage	120	Placebo (30), ARB (32), BB (30), both (28)	51 ± 10	100%	2	Candesartan (8–32 mg Qd), metoprolol succinate (50–100 mg Qd)	14	EOT (14)	LVEF, adverse events	MRI (CMR echo)	63.1, 62.5, 63.3, 61.7
Elitok et al. 2014 [25]	Anthracycline	NA	80	Control (40), BB (40)	52.9 ± 11	100%	6	Carvedilol (12.5 mg Qd)	6	EOT (180)	LVEF, FS, LV dimensions	Echo	65 ± 4.5, 65 ± 6.1
Kaya et al. 2013 [26]	Anthracycline	NA	45	Placebo (18), BB (27)	50.5 ± 11	100%	NA	Nebivolol (5 mg Qd)	6	6 (180)	LVEF	Echo	66.6 ± 5.5, 65.6 ± 4.8

*ACEI *angiotensin-converting enzyme inhibitor*, ARB *angiotensin receptor blocker*, BB *beta-blocker*, BNP *brain natriuretic peptide*, Bid *bis in die*, FS *fractional shortening*, GLS *global longitudinal strain of the LV*, LA *left atrium*, LV *left ventricle*, LVEF *left ventricular ejection fraction*, NA *not available*, PAP *pulmonary artery pressure*, Qd *quaque die*, SRS *the strain rate of the LV systolic function *,TNI *troponin I

### Search strategy

From their inception through May 11, 2022, we searched PubMed, EMBASE, Cochrane Library, and Web of Science without regard to language or abstract requirements. Two authors (YG and JJ) were the only ones for whom the search was allowed. In addition, we searched past systematic reviews for any references to RCTs that were not returned by our own search. Reviews, in vitro experiments, and animal research were not included. The intended studies were RCTs comparing the effects of anthracyclines, trastuzumab, and mono- or combination therapy with an ACEI, ARB, or BB on LVEF in comparison to placebo. The search terms were anthracycline*/Doxorubicin*/Adriamycin*/Daunorubicin*/Epirubicin*/Trastuzumab/beta blocker/ACEI/ARB et al. Supplemental Table 2 contains the PubMed search methodology.

### Eligibility criteria of articles

We incorporated published RCTs comparing ACEI, ARB, BB, or both with placebo. RCTs involving breast cancer patient enrollment qualified as studies for inclusion. We counted each intervention separately for trials that included more than two comparisons. Studies were to include data on adverse events and LVEF in percent (%) before and after breast cancer treatment. A multi-gated acquisition (MUGA) scan, magnetic resonance imaging (MRI), or echocardiography might have been used to determine the LVEF. Studies that were not randomized, looked at different tumor kinds, or in which the BC group’s outcomes could not be examined separately were eliminated.

The titles and abstracts of the retrieved papers were examined independently by two reviewers (YG and RTW). The complete texts of trials that might be considered eligible were studied in order to make this determination. Discussion was used to settle any disputes, and a third reviewer (HL) was contacted if necessary.

### Data extraction

Using standardized forms, two reviewers (YG and JJ) independently retrieved data from accepted papers. A third reviewer (HL) settled any differences. Publication data were among the information that was extracted (e.g., publication year, authors), participant data (e.g., number, female sex, age, primary study drug), intervention data (e.g., drug, duration, duration of ACEI/ARB or BB), and outcome data (e.g., mean baseline EF, mean final EF, type of LVEF measurement).

### Risk of bias assessment

Using version 2 of Cochrane’s revised tool to assess the risk of bias in randomized trials (RoB 2), two reviewers (YG and JJ) independently evaluated the risk of bias [10] (see Supplementary Table 3). Any discrepancies were discussed with a third reviewer (RTW) before being decided. Based on the randomization method, variations from the intended intervention, missing outcome data, measurement of the outcome, and choice of the report result, we assessed the likelihood of bias.

### Assessment of evidence quality

The quality of the evidence was evaluated using the GRADE (grading of recommendations assessment, development, and evaluation) assessment [11]. Each key outcome’s finding table was summarized and presented.

### Statistics

The outcome data on the results are shown as mean difference (MD) with standard deviation (SD) or with associated 95% confidence intervals (95% CIs) of LVEF during the follow-up period. If MD ± SD was not presented, it was computed using the VassarStats calculator based on the median, range, and number of cases. Considering differences in the staging of breast cancer, treatment duration, and demographic characteristics in different studies, we performed meta-analyses of RCT data with a random effects model. Statistical analysis was performed using Stata 15 and R software 4.2.1. We used a frequentist meta-analysis to produce forest plots and estimate the *I*^2^ statistic along with a 95% confidence interval, which indicates the percentage of the variability that is not due to random errors. This approach will provide a heterogeneity assessment.

## Results

### Search results

**Fig. 1:**
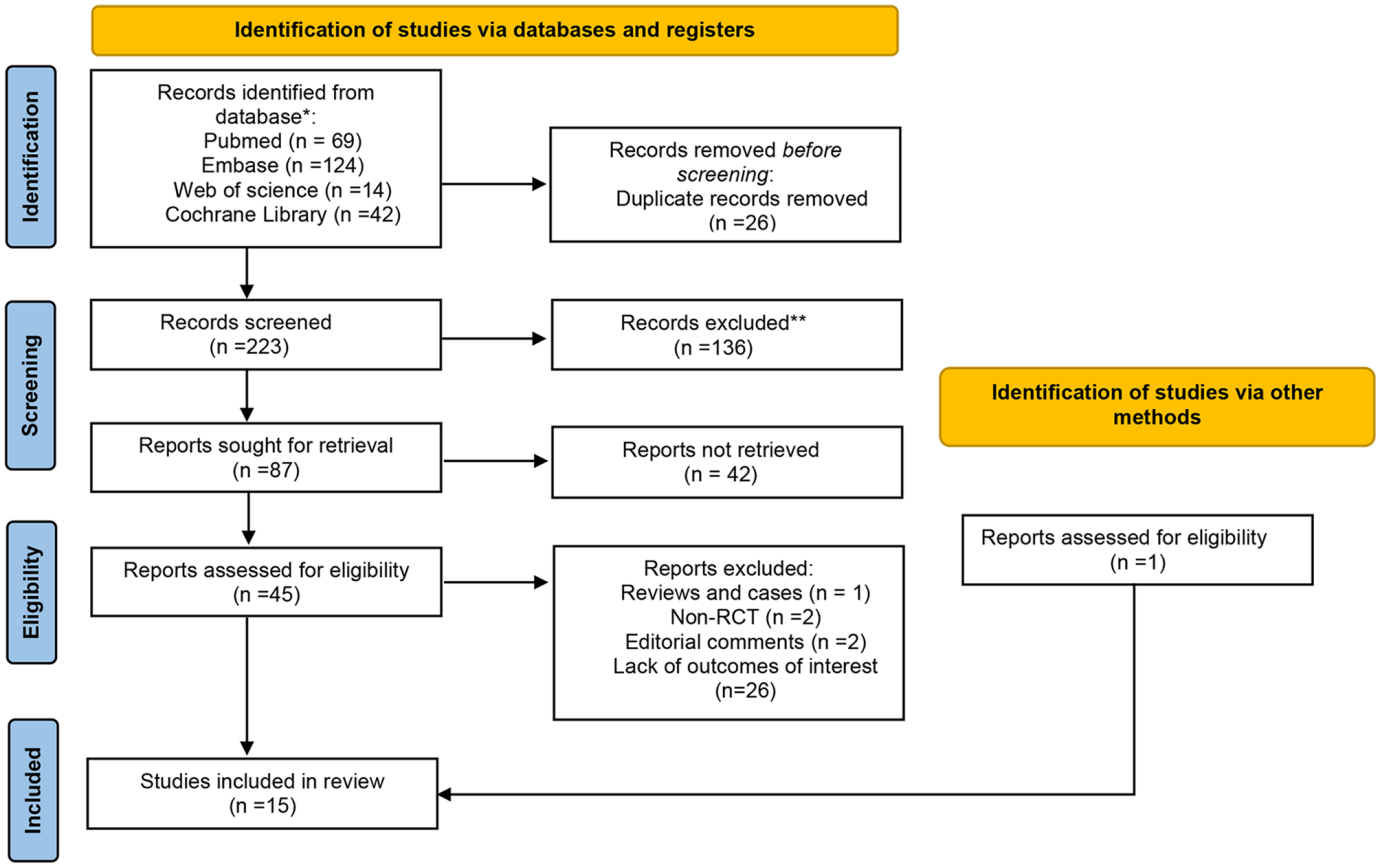
The PRISMA search strategy flow chart

A total of 249 studies were found in our preliminary search in PubMed, Embase, Cochrane Library, and Web of Science. After removing duplicates, 223 studies were assessed for eligibility. Additionally, one study was included from the references, through which we reviewed previous systematic reviews. Fifteen random clinical studies [12–26] with a total of 1977 patients were included in the analysis. Figure 1 depicts the PRISMA search strategy.

### Study characteristics

We included 1977 female patients with breast cancer. Eight studies [12, 15–17, 20, 22, 23, 25] concerned patients with early-stage cancer. Only one study [13] was concerned with locally advanced breast cancer, and six studies [14, 18, 19, 21, 25, 26] did not limit the patients’ cancer stage. All patients received anthracycline or trastuzumab. The mean age varied from 39.9 to 57.4 years between the studies. The baseline LVEF mean ranged between 54.3 and 68.7%. Eight studies reported adverse events [13, 14, 17, 19–21, 23, 25]. The characteristics of the 15 studies are reported in Table 1.

### Outcomes

**Fig. 2:**
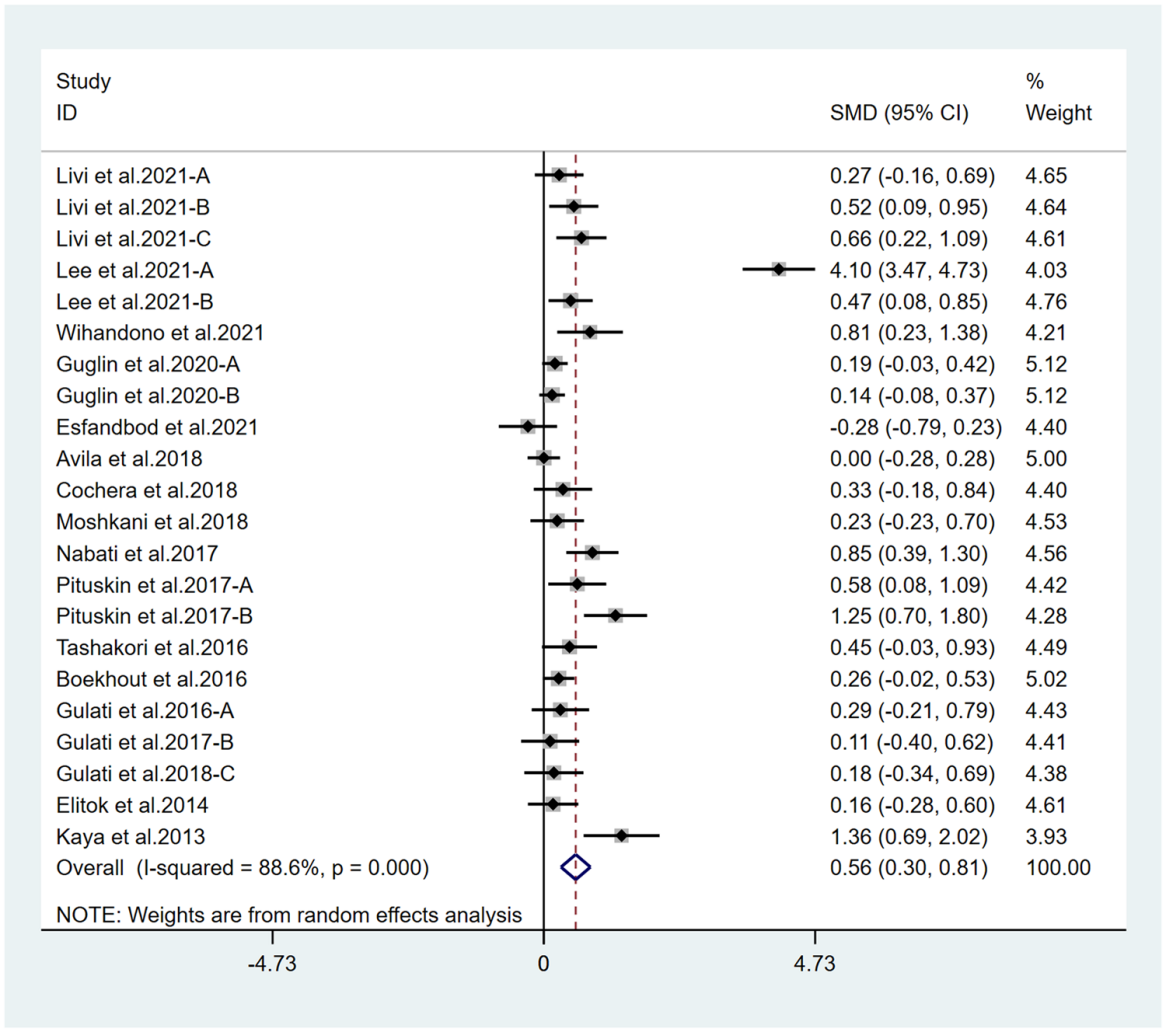
Primary outcome. Meta-analysis of the impact of concomitant treatment with ACEIs, ARBs, and BBs compared with placebo on left ventricular ejection fraction in patients treated with anthracyclines or trastuzumab. SMD, standardized mean difference; CI, confidence interval

**Fig. 3:**
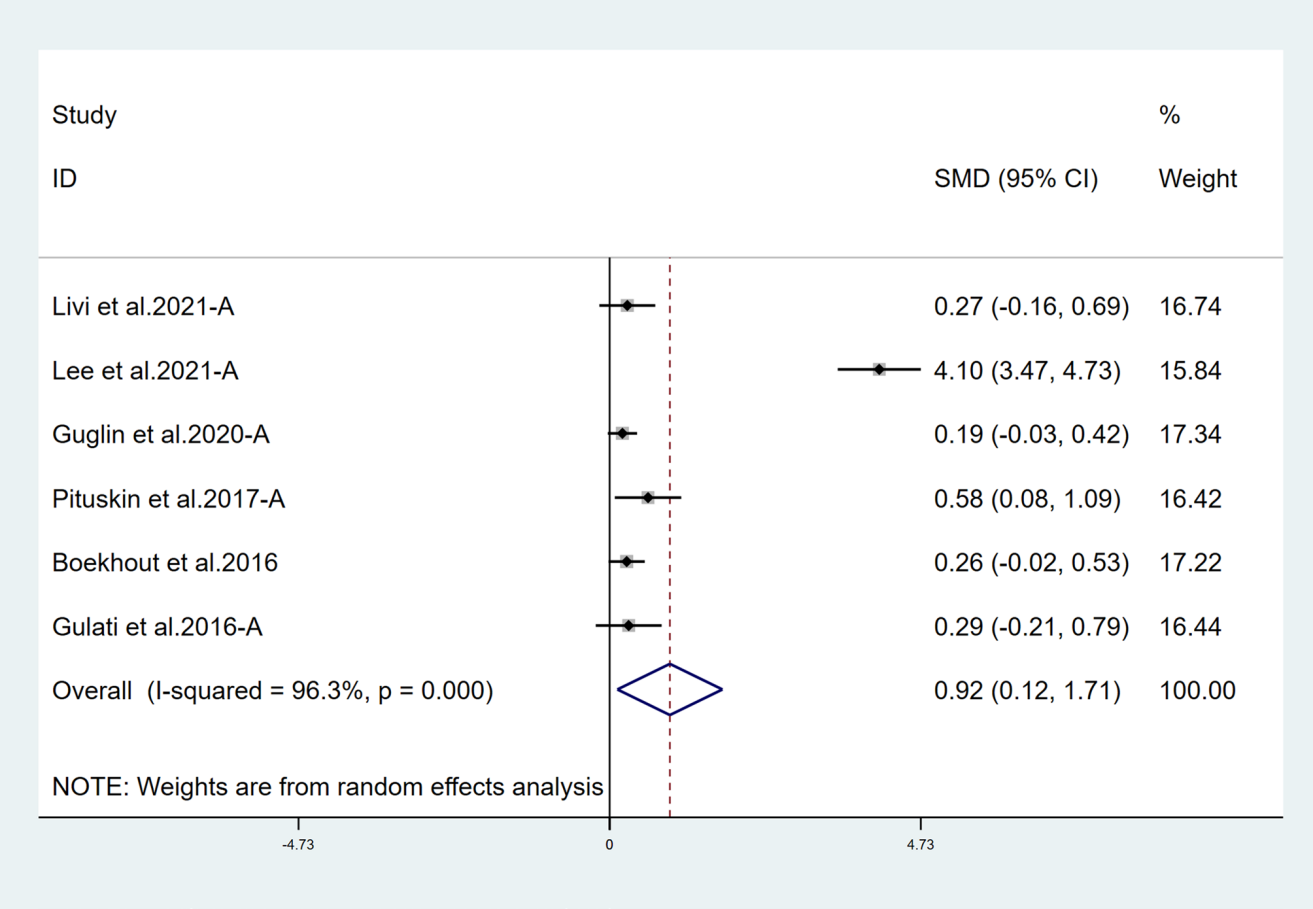
Meta-analysis of the impact of concomitant treatment with ACEIs or ARBs alone compared with placebo on left ventricular ejection fraction in patients treated with anthracyclines or trastuzumab. SMD, standardized mean difference; CI, confidence interval

**Fig. 4:**
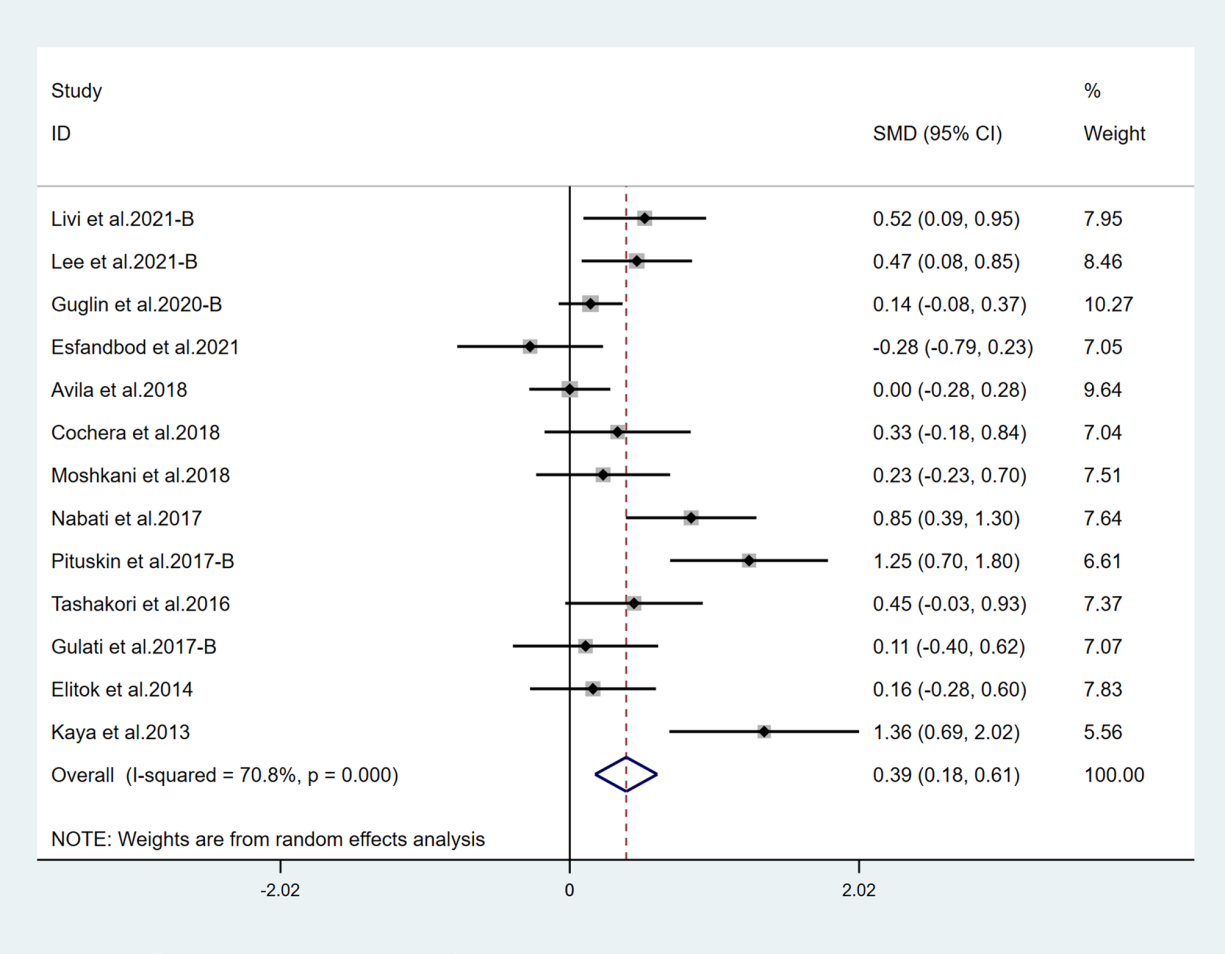
Meta-analysis of the impact of concomitant treatment with BBs alone compared with placebo on left ventricular ejection fraction in patients treated with anthracyclines or trastuzumab. SMD, standardized mean difference; CI, confidence interval

**Fig. 5:**
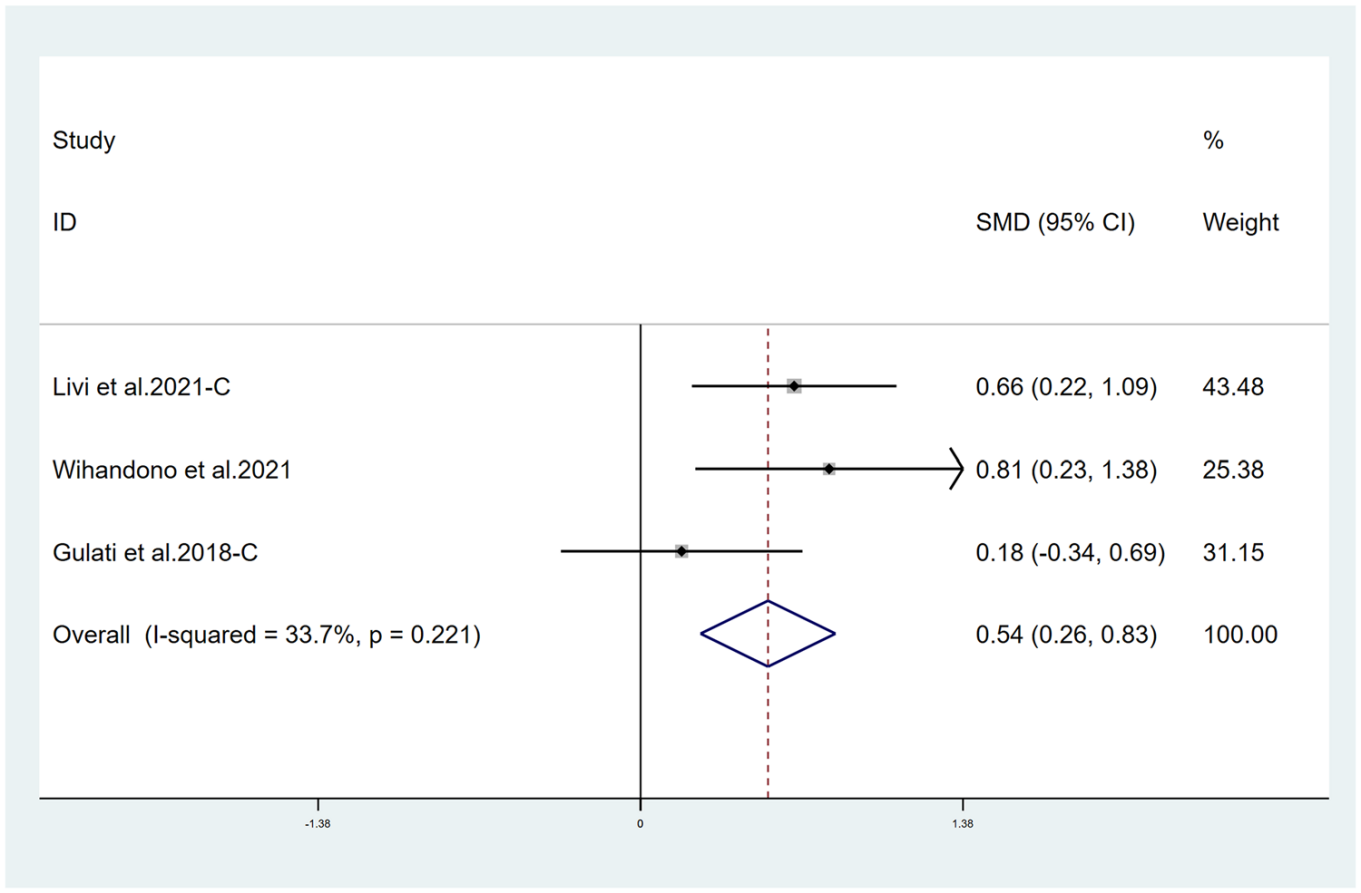
Meta-analysis of the impact of concomitant treatment with ACEIs or ARB combined with BB compared with placebo on left ventricular ejection fraction in patients treated with anthracyclines or trastuzumab. SMD, standardized mean difference; CI, confidence interval

Fifteen studies reported LVEF and suggested a statistically significant higher LVEF in the treatment group (*χ*^2^ = 184.75, *I*^2^ = 88.6%, *p* = 0.000; SMD 0.556, 95% CI 0.299 to 0.813) (Fig. 2). Both ACEI and ARB are renin–angiotensin–aldosterone system (RAAS) inhibitors, so we combined them for analysis. Six studies reported LVEF in ACEI/ARB alone and suggested a statistically significant higher LVEF in the treatment group (*χ*^2^ = 136.62, *I*^2^ = 96.3%, *p* = 0.000; SMD 0.915, 95% CI 0.116 to 1.714) (Fig. 3). Thirteen studies also suggested that BB therapy preserved LVEF significantly better than placebo (*χ*^2^ = 41.03, *I*^2^ = 70.8%, *p* = 0.000; SMD 0.393, 95% CI 0.177 to 0.610) (Fig. 4). Three studies reported LVEF in ACEI/ARB combined with BB and suggested a statistically significant higher LVEF in the treatment group (*χ*^2^ = 3.02, *I*^2^ = 33.7%, *p* = 0.221; SMD 0.543, 95% CI 0.256 to 0.832) (Fig. 5).

Furthermore, ACEI/ARB or BB therapy suggested a statistically significant higher LVEF in the treatment group (*χ*^2^ = 155.43, *I*^2^ = 91.0%, *p* = 0.000; SMD 0.680, 95% CI 0.285 to 1.075) during anthracycline therapy (see Supplementary Fig. 1). During trastuzumab therapy, five studies also suggested a statistically significant higher LVEF in the treatment group (*χ*^2^ = 19.56, *I*^2^ = 69.3%, *p* = 0.003; SMD 0.300, 95% CI 0.062 to 0.538) (see Supplementary Fig. 2).

### Adverse events

Eight studies reported adverse events [13, 14, 17, 19–21, 23, 24]. In seven of them, no adverse events occurred in both the test or control groups [13, 14, 17, 20, 21, 23, 24]. Avila et al. reported nine patients stopped the drug due to side effects, 6 (6.2%) in the placebo group and 3 (3.1%) in the carvedilol group (*p* = 0.30) [19].

### Sensitivity analysis

Sensitivity analysis was conducted to investigate possible sources of heterogeneity by removing the data in sequence. The result indicated that the meta-analyses outcomes were stable, as shown in Supplementary Fig. 3.

### Risk of bias

A total of 5 out of 15 studies were assessed to have a high risk of bias in overall bias. The overall trend in these assessments was the randomization process and the blinding of both participants and investigators (Supplementary Fig. 4). Five included studies were assessed as high risk in terms of deviations from intended interventions, for their open-label and placebo-controlled design. As noted by the authors, this can be partly explained by ethical considerations and informed consent to include late-phase breast cancer patients.

### Overall quality of the evidence

Summary of the finding table was used to show the overall quality of the evidence for the primary outcome, LVEF. To present the results more intuitively, we summarize direct proof of all types of comparisons related to these outcomes in one table (Supplementary Table 4). The reasons to downgrade the quality of evidence are limitations of methodological quality of included trials according to the risk of bias assessment, statistical heterogeneity among included trials, and the small sample size. There were 13 (59.09%) moderate-quality evidence and 9 (40.91%) low-quality evidence.

## Discussion

Based on the analysis of key outcome indicators, this study conducted a meta-analysis of 15 RCTs and found that prophylactic treatment with BBs and/or ACEI/ARB greatly enhances LVEF in comparison to anthracyclines or trastuzumab. The reduction of ventricular remodeling and anti-oxidative stress by these two medicines may be connected to the protective mechanism [29]. The lack of a substantial increase in LVEF in four studies using ACEI/ARB alone suggests that the favorable findings may be attributable to this early study [14]. The subgroup analysis’s findings also suggested that ACEI/ARB/BBs might offer protection against cardiotoxicity brought on by both anthracyclines and trastuzumab.

In recent years, more and more studies have focused on the cardiotoxicity of trastuzumab in combination with anthracyclines and have attempted to reduce it [30]. Doxorubicin liposomal has proven effective in reducing the harmful effects that doxorubicin can have on the heart, and new studies are still ongoing and gaining ground [31]. In many previous studies, the cardiotoxicity associated with trastuzumab was assumed to be transient and reversible with drug interruption or discontinuation. Recent data has partially revised this assumption, reporting a non-negligible incidence of long-term cardiotoxicity [32].

The recommended initial medications for treating heart failure are ACEI/ARB and BB. Research has indicated that ACEI/ARB and BB may have a positive impact on reducing the long-term health risks associated with anthracycline-induced cardiac dysfunction [12, 16]. Previous clinical studies have examined the treatment of doxorubicin-induced cardiotoxicity, but the patients involved had tumors other than breast cancer [33, 34]. Lewinter et al. [7], Elghazawy et al. [8], and Jeyaprakash et al. [35] published a similar meta-analysis. Elghazawy et al. not only focused on randomized controlled trials, but also included observational studies. However, they did not extract the breast cancer subgroup indicators from the original study. According to Lewinter et al., the use of ACEI/ARB therapy, regardless of whether it is combined with anthracycline or trastuzumab therapy, resulted in a slightly higher LVEF compared to a placebo, although the difference was not statistically significant. Jeyaprakash et al.’s study found that cardioprotective agents did not differ in their effect on LVEF according to Bayesian analysis. However, frequentist analysis suggested that ACEI/ARB and BB may offer significant cardio-protection. The inconsistency with the results of previous studies may be the different types and stages of the tumor and low-quality articles. The results still need to be further validated by high-quality RCT.

In clinical practice, it is crucial to establish multidisciplinary teams where cardiologists and oncologists work together to give the best possible care to oncology patients undergoing treatment with cardiotoxic agents. Based on our findings, there is no evidence to suggest that ACEI/ARBs are more effective than BBs in preventing a decrease in LVEF among patients undergoing breast cancer treatment.

This study also has some limitations. More substantial, high-quality, randomized, double-blind controlled trials are required to assess the clinical efficacy of beta-blockers and angiotensin receptor antagonists in the prevention of anthracycline-induced early cardiotoxicity, as the majority of the included studies only evaluated a small number of patients, and the heterogeneity of the unknown cause remains unexplained. Meanwhile, total heterogeneity ranged from moderate to high. The causes of the variations between the RCTs were not identified. Meanwhile, the results may be impacted by the method used to measure LVEF. However, the lack of sufficient studies makes it difficult to conduct a subgroup analysis on this topic.

Future research will also investigate the efficacy and safety of new medications as well as complementary therapies. For example, sacubitril/valsartan, an angiotensin receptor-neprilysin inhibitor, outperforms standard therapy in the treatment of heart failure with reduced ejection fraction, but further research is required to determine its cardioprotective effects in the context of cardio-oncology. There is a randomized, placebo-controlled, double-blind, multi-center clinical trial [36] to see if sacubitril/valsartan given concurrently with early breast cancer therapy regimens comprising anthracyclines, with or without trastuzumab, may prevent cardiac dysfunction. Wang et al. [37], among others, have found that several herbal monomers have cardioprotective benefits in animal experiments.

## Conclusion

When compared to placebo, ACEI/ARB and BB treatments can shield breast cancer patients from cardiotoxicity during trastuzumab and anthracycline-containing regimens, suggesting that both are helpful.

### Supplementary Information

Below is the link to the electronic supplementary material.

Supplementary Fig. 1: Meta-analysis of the impact of concomitant treatment with ACEI/ARBs and BBs compared with placebo on left ventricular ejection fraction in patients treated with anthracyclines as a primary drug. SMD, standardized mean difference; CI, confidence interval (TIF 518 kb)

Supplementary Fig. 2: Meta-analysis of the impact of concomitant treatment with ACEI/ARBs and BBs compared with placebo on left ventricular ejection fraction in patients treated with trastuzumab as primary drug. SMD, standardized mean difference; CI, confidence interval (TIF 3471 kb)

: Sensitivity-analysis of the analysis, given name study is omitted; CI, confidence interval (TIF 9110 kb)

Risk of bias summary: review authors' judgments about each risk of bias item for each included study. Fig. 9B Risk of bias graph: review authors' decisions about each risk of bias item presented as percentages across all included studies. (TIF 417 kb)

Supplementary table 1: The PRISMA Checklist 2020 (DOCX 31 kb)

Supplementary table 2: The search strategy in Pubmed (DOC 35 kb)

Supplementary table 3: Version 2 of the Cochrane tool for assessing risk of bias in randomized trial, RoB2 (PDF 560 kb)

Supplementary table 4: The summary of finding tables for LVEF in patients who are receiving cardiotoxic therapy (DOCX 17 kb)

## Data Availability

Not applicable.
