# Supplementary figures and images for: ACEI/ARB and beta-blocker therapies for preventing cardiotoxicity of antineoplastic agents in breast cancer: a systematic review and meta-analysis
Source: Heart Fail Rev. 2023 Jul 7;28(6):1405–15. doi: 10.1007/s10741-023-10328-z (PMC10575808; doi:10.1007/s10741-023-10328-z)

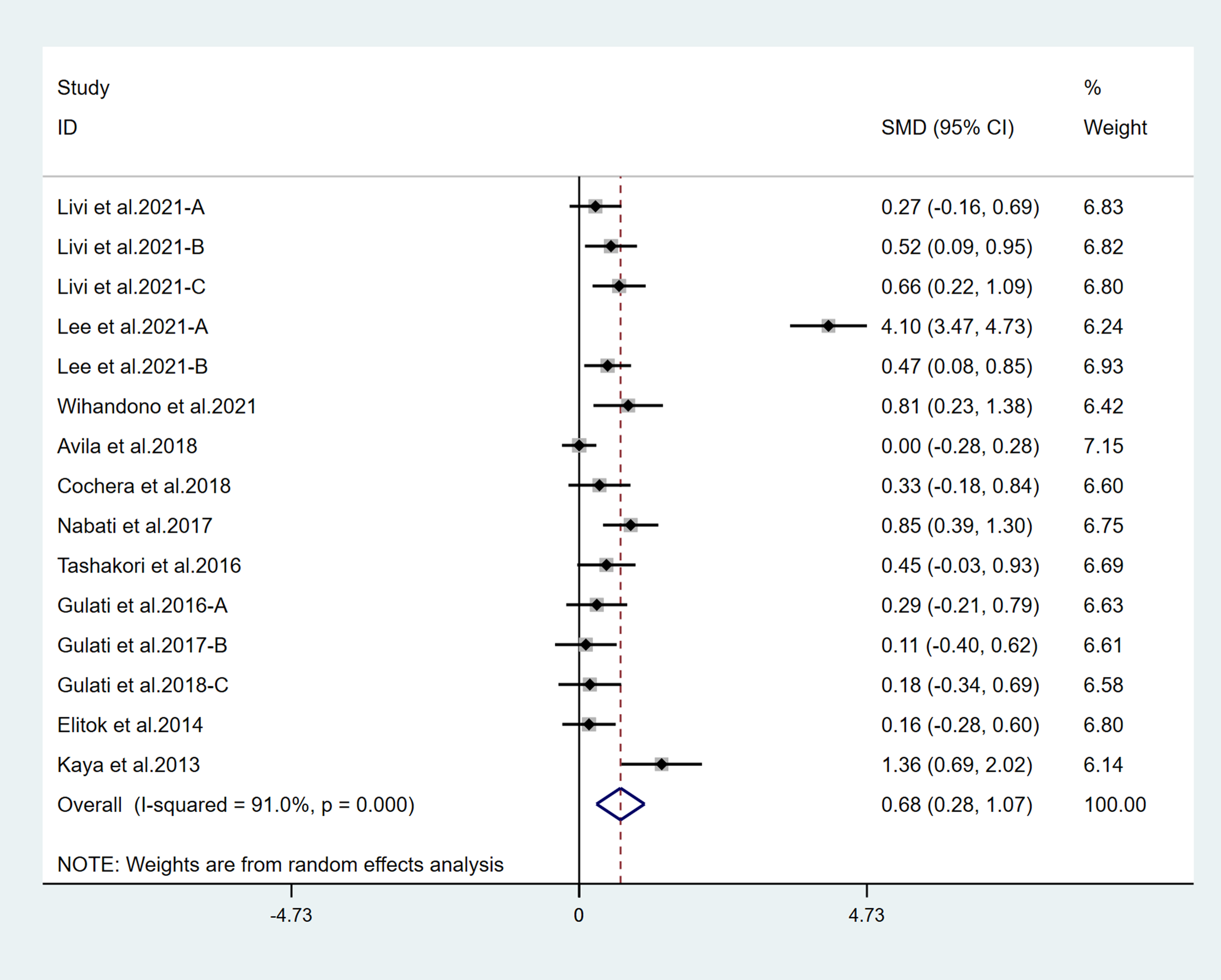

Supplement: Supplementary file 1 — Supplementary Fig. 1: Meta-analysis of the impact of concomitant treatment with ACEI/ARBs and BBs compared with placebo on left ventricular ejection fraction in patients treated with anthracyclines as a primary drug. SMD, standardized mean difference; CI, confidence interval (TIF 518 kb) [file 10741_2023_10328_MOESM1_ESM.tif]

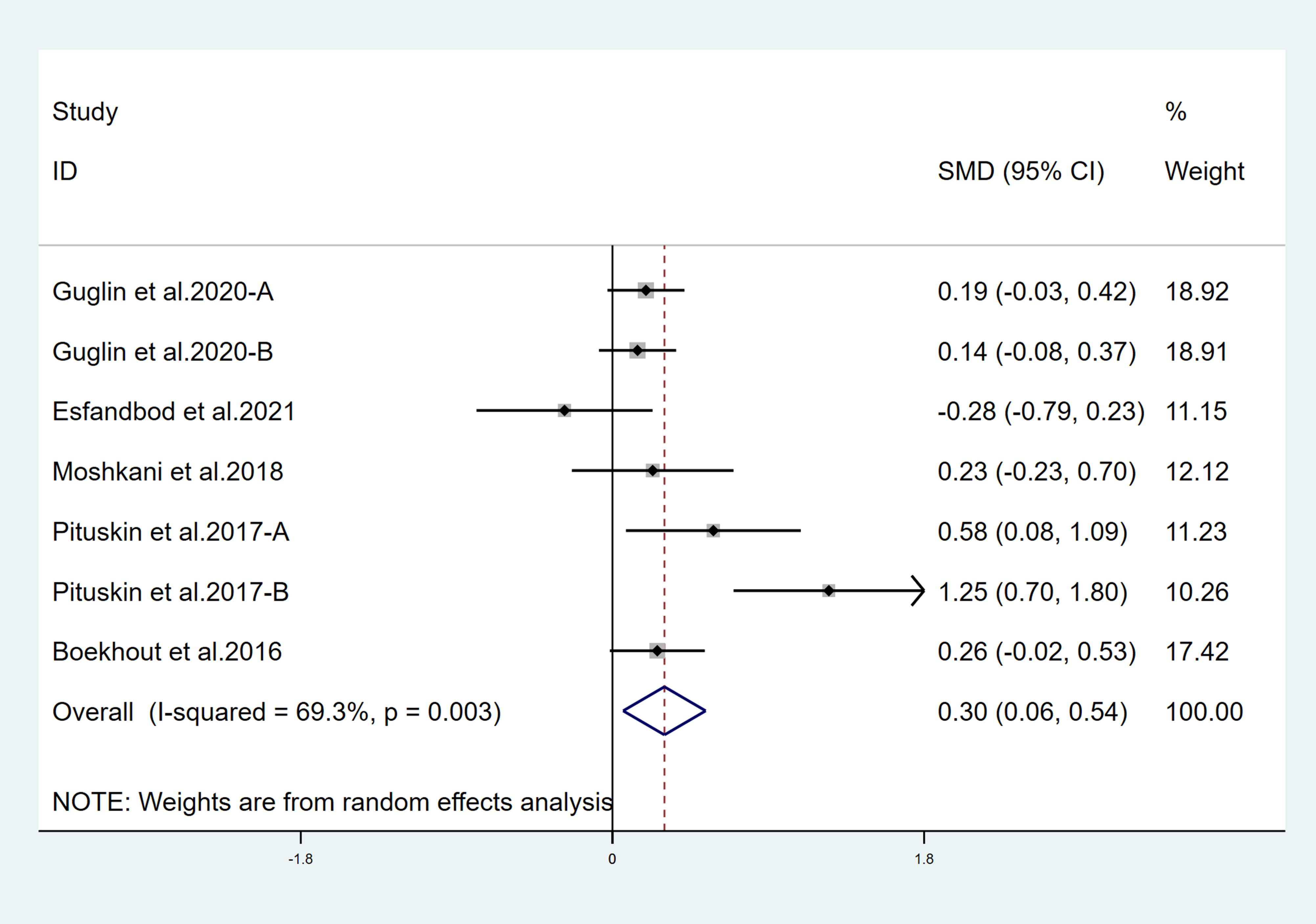

Supplement: Supplementary file 2 — Supplementary Fig. 2: Meta-analysis of the impact of concomitant treatment with ACEI/ARBs and BBs compared with placebo on left ventricular ejection fraction in patients treated with trastuzumab as primary drug. SMD, standardized mean difference; CI, confidence interval (TIF 3471 kb) [file 10741_2023_10328_MOESM2_ESM.tif]

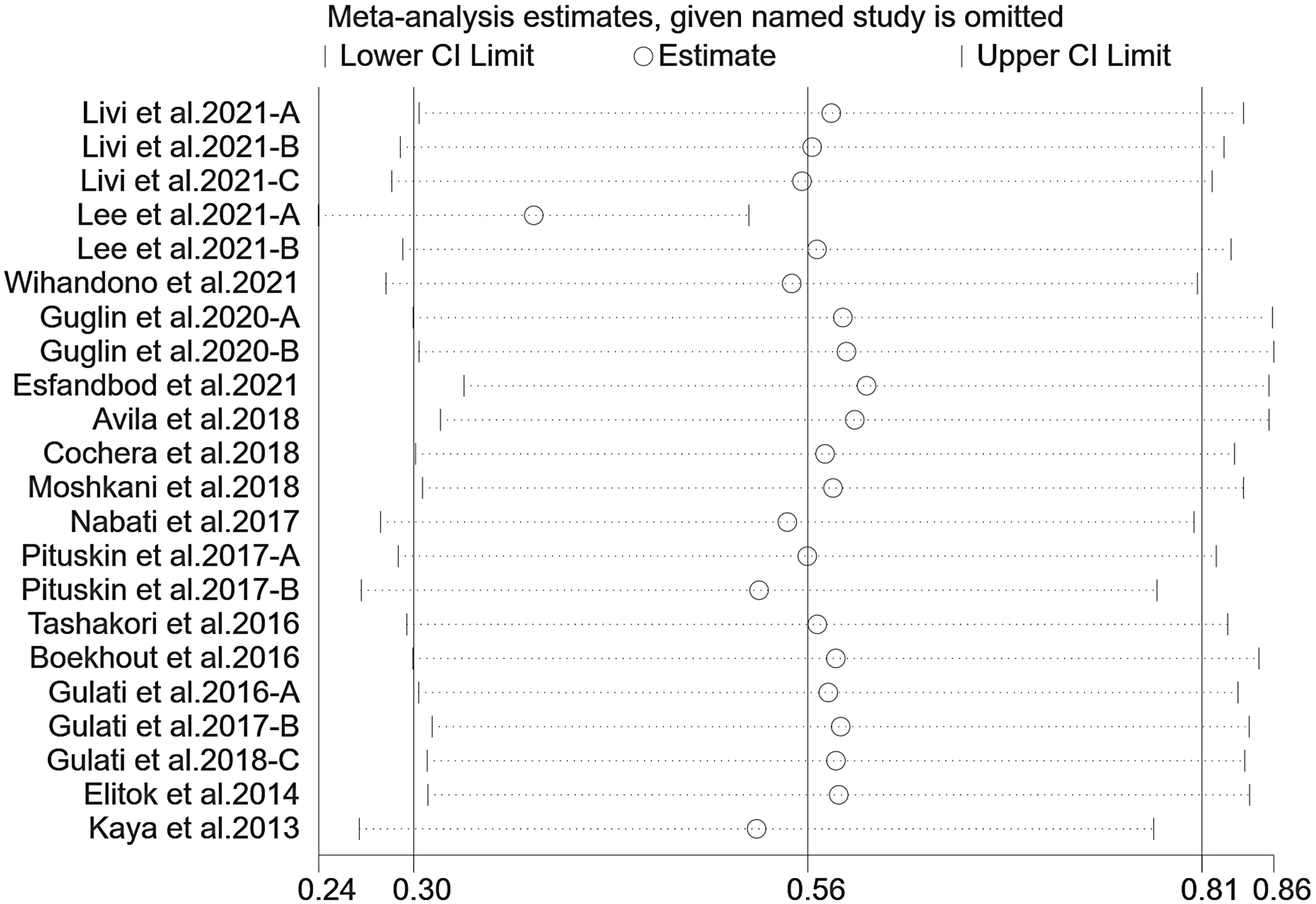

Supplement: Supplementary file 3 — : Sensitivity-analysis of the analysis, given name study is omitted; CI, confidence interval (TIF 9110 kb) [file 10741_2023_10328_MOESM3_ESM.tif]

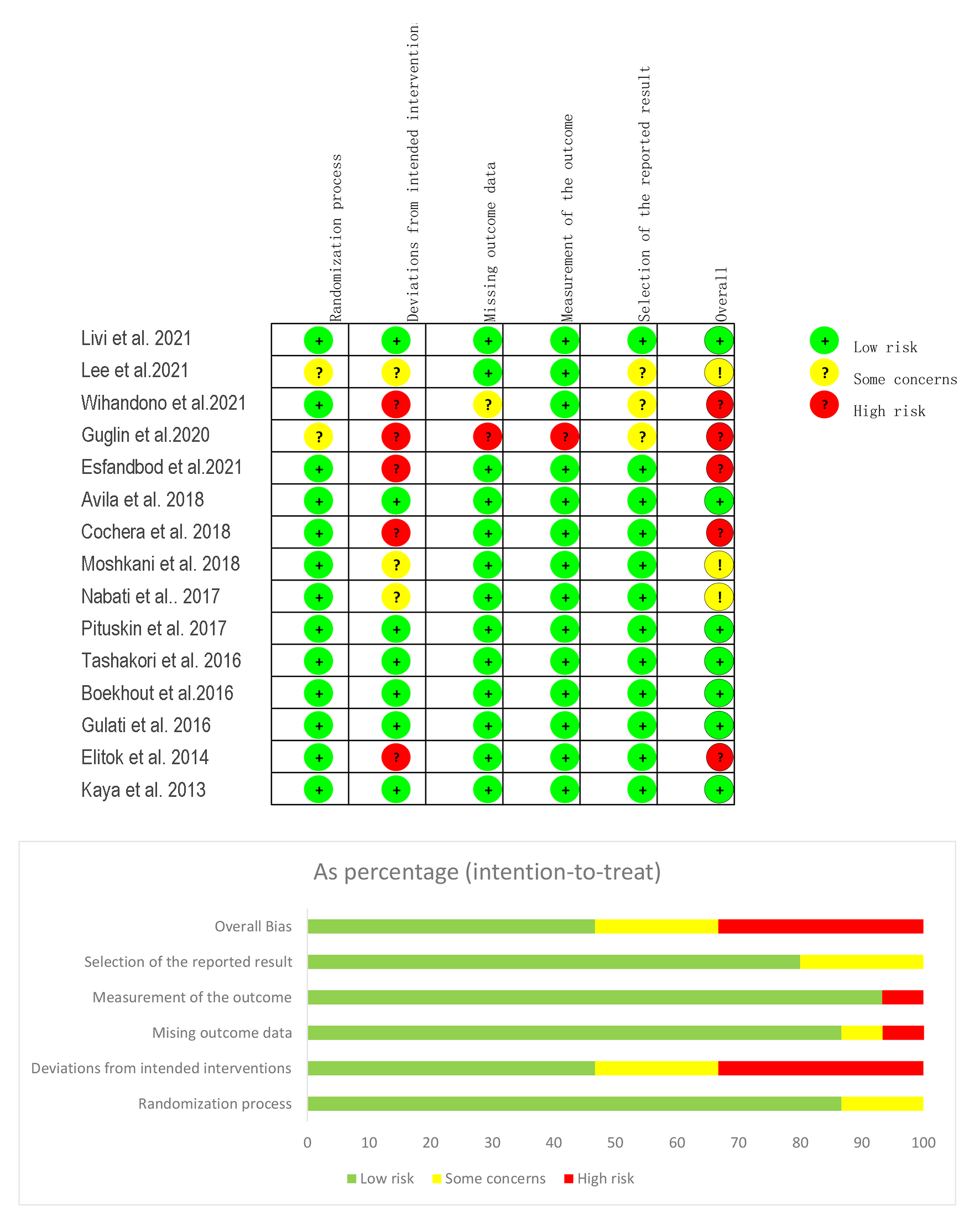

Supplement: Supplementary file 4 — Risk of bias summary: review authors' judgments about each risk of bias item for each included study. Fig. 9B Risk of bias graph: review authors' decisions about each risk of bias item presented as percentages across all included studies. (TIF 417 kb) [file 10741_2023_10328_MOESM4_ESM.tif]
