# Supplementary material for: ACEI/ARB and beta-blocker therapies for preventing cardiotoxicity of antineoplastic agents in breast cancer: a systematic review and meta-analysis
Source: Heart Fail Rev. 2023 Jul 7;28(6):1405–15. doi: 10.1007/s10741-023-10328-z (PMC10575808; doi:10.1007/s10741-023-10328-z)
Supplement: Supplementary file 6 — Supplementary table 2: The search strategy in Pubmed (DOC 35 kb) [file 10741_2023_10328_MOESM6_ESM.doc]

**Search terms in PubMed May 11, 2022**

(((((((beta blocker) OR (ACEI)) OR (beta-blocker)) OR (β blocker)) OR (β-blocker)) OR (ARB)) AND (((((((anthracycline*) OR (Doxorubicin*)) OR (Adriamycin*)) OR (Daunorubicin*)) OR (Epirubicin *)) OR (Trastuzumab)) OR (Herceptin))) AND ((breast cancer) OR (breast cancer[MeSH Terms]))

| Search number | Query | Results | Time |
| --- | --- | --- | --- |
| 6 | ((((((((anthracycline*) OR (Doxorubicin*)) OR (Adriamycin*)) OR (Daunorubicin*)) OR (Epirubicin *)) OR (Trastuzumab)) OR (Herceptin)) AND ((breast cancer) OR (breast cancer[MeSH Terms]))) AND (((((beta blocker) OR (ACEI)) OR (beta-blocker)) OR (β blocker)) OR (β-blocker)) | 69 | 12:06:30 |
| 5 | (((((((((anthracycline*) OR (Doxorubicin*)) OR (Adriamycin*)) OR (Daunorubicin*)) OR (Epirubicin *)) OR (Trastuzumab)) OR (Herceptin)) AND ((breast cancer) OR (breast cancer[MeSH Terms]))) AND (((((beta blocker) OR (ACEI)) OR (beta-blocker)) OR (β blocker)) OR (β-blocker))) AND (((((Randomized) OR (randomised)) OR (random)) OR (RCT)) OR (randomly)) | 28 | 11:48:00 |
| 4 | ((((Randomized) OR (randomised)) OR (random)) OR (RCT)) OR (randomly) | 1,563,969 | 11:47:03 |
| 3 | ((((beta blocker) OR (ACEI)) OR (beta-blocker)) OR (β blocker)) OR (β-blocker) | 101,831 | 11:45:59 |
| 2 | (breast cancer) OR (breast cancer[MeSH Terms]) | 463,910 | 11:44:34 |
| 1 | ((((((anthracycline*) OR (Doxorubicin*)) OR (Adriamycin*)) OR (Daunorubicin*)) OR (Epirubicin *)) OR (Trastuzumab)) OR (Herceptin) | 116,002 | 11:43:27 |
| 0 | Clipboard | 28 | 12:08:03 |
