# Supplementary material for: ACEI/ARB and beta-blocker therapies for preventing cardiotoxicity of antineoplastic agents in breast cancer: a systematic review and meta-analysis
Source: Heart Fail Rev. 2023 Jul 7;28(6):1405–15. doi: 10.1007/s10741-023-10328-z (PMC10575808; doi:10.1007/s10741-023-10328-z)
Supplement: Supplementary file 8 — Supplementary table 4: The summary of finding tables for LVEF in patients who are receiving cardiotoxic therapy (DOCX 17 kb) [file 10741_2023_10328_MOESM8_ESM.docx]

Supplementary table 4 The summary of finding tables for LVEF in patients who are receiving cardiotoxic therapy

| **Quality assessment** | | | | | | | **No of patients** | | **Effect** | | **Quality** | **Importance** |
| --- | --- | --- | --- | --- | --- | --- | --- | --- | --- | --- | --- | --- |
|  |  |  |  |  |  |  |  |  |  |  |  |  |
| **No of comparisons** | **Design** | **Risk of bias** | **Inconsistency** | **Indirectness** | **Imprecision** | **Other considerations** | **ACEI/ARB and Beta-Blocker Therapies** | **Control** | **Relative (95% CI)** | **Absolute** |  |  |
| **ACEI/ARB/BB vs. placebo (Better indicated by higher values)** | | | | | | | | | | | | |
| 22 | randomized trials | serious^1^ | very serious^2^ | no serious indirectness | no serious imprecision | none | 1211 | 1127 | - | SMD 0.56 higher (0.30 to 0.81 higher) |  VERY LOW |  |
| **ACEI/ARB vs. placebo (Better indicated by higher values)** | | | | | | | | | | | | |
| 6 | randomized trials | no serious risk of bias | very serious^2^ | no serious indirectness | no serious imprecision | none | 452 | 402 | - | SMD 0.92 higher (0.12 to 1.71 higher) |  LOW |  |
| **BB vs. placebo (Better indicated by higher values)** | | | | | | | | | | | | |
| 13 | randomized trials | serious^1^ | no serious inconsistency^2^ | no serious indirectness | no serious imprecision | none | 662 | 628 | - | SMD 0.393 higher (0.177 to 0.610 higher) |  MODERATE |  |
| **ACEI/ARB combined with BB vs. placebo (Better indicated by higher values)** | | | | | | | | | | | | |
| 3 | randomized trials | no serious risk of bias | no serious inconsistency | no serious indirectness | serious^3^ | reporting bias^4^ | 97 | 97 | - | SMD 0.543 higher (0.186 to 0.900 higher) |  LOW |  |
| **During anthracycline therapy (Better indicated by higher values)** | | | | | | | | | | | | |
| 15 | randomized trials | no serious risk of bias | very serious^2^ | no serious indirectness | no serious imprecision | none | 664 | 591 | - | SMD 0.680 higher (0.285 to 1.075 higher) |  LOW |  |
| **During trastuzumab therapy (Better indicated by higher values)** | | | | | | | | | | | | |
| 7 | randomized trials | no serious risk of bias | serious^5^ | no serious indirectness | no serious imprecision | none | 547 | 536 | - | SMD 0.300 higher (0.062 to 0.538 higher) |  MODERATE |  |

^1^ There were serious limitations of the methodological quality of included trials according to the risk of bias assessment.
^2^ There were very serious statistical heterogeneity among included trials. 
^3^ There was serious imprecision.
^4^ There was serious publication bias.

^5^ There was serious statistical heterogeneity among included trials.
